# Can an app supporting psoriasis patients improve adherence to topical treatment? A single-blind randomized controlled trial

**DOI:** 10.1186/s12895-018-0071-3

**Published:** 2018-02-07

**Authors:** Mathias Tiedemann Svendsen, Flemming Andersen, Kirsten Hammond Andersen, Klaus Ejner Andersen

**Affiliations:** 1 Department of Dermatology and Allergy Centre, Odense University Hospital, Kløvervænget 15, 5000 Odense C, Denmark; 2 Centre for Innovative Medical Technology, Institute of Clinical Research, University of Southern Denmark, Odense C, Denmark; 3 Odense Patient data Explorative Network (OPEN), Odense University Hospital, Odense, Denmark; 4 Department of Clinical Research, University of Southern Denmark, Odense, Denmark; 5 Dermatological Investigations Scandinavia (DIS), University of Southern Denmark, Odense C, Denmark

**Keywords:** Psoriasis, Adherence, App, Smartphone, Electronic monitor, Randomized controlled trial (RCT)

## Abstract

**Background:**

Topical corticosteroid or corticosteroid/calcipotriol preparations are recommended first-line topical treatments of psoriasis, but a main cause for the lack of efficacy of topical treatments is considered low rates of adherence to topical drugs. Patient support by the use of applications (apps) for smartphones is suggested to improve medical adherence.

**Methods/design:**

*Design:* An investigator-initiated, single-center, single-blind, parallel-group, phase-4 clinical superiority randomized controlled trial (RCT). *Participants:* 134 patients 18 to 75 years of age with mild-to-moderate psoriasis, who are capable of reading English language, own a smartphone, and are candidates for the study drug calcipotriol and betamethasone dipropionate (Cal/BD) cutaneous foam once daily prn (pro re nata). *Intervention:* A 28-day adherence-supporting app providing compulsory daily treatment reminders that pop-up on the smartphone screen with a short alert sound. The app synchronizes through Bluetooth® to an electronic monitor (EM) attached to the medication canister. The EM contains a chip registering the amount of foam, day and time the patient use the foam dispenser. The information is displayed in a diary that shows the amount of Cal/BD cutaneous foam used and the number of applied treatment sessions. The app has an optional diary with the patient’s rating of symptoms. *Non-intervention:* Use of Cal/BD cutaneous foam and EM without the app. All participants are prescribed Cal/BD cutaneous foam prn for the entire study period.

*Primary outcome obtained in week 4:* rates of adherence measured by patient report, weight of medication canisters, and number of treatment sessions measured by the EM. *Secondary outcomes obtained at baseline, weeks 4, 8, and 26:* Lattice System Physician’s Global Assessment (LS-PGA) and Dermatology Quality of Life Index (DLQI).

**Discussion:**

This trial tests of whether an app can improve rates of adherence to a topical antipsoriatic drug. If the app improves rates of adherence and reduces the burden of psoriasis in a clinically significant way, the app could easily be implemented as a standard routine of care in the clinic.

**Trial registration:**

NCT02858713, registered on August 3, 2016. EudraCT number 2016–002143-42.

**Electronic supplementary material:**

The online version of this article (10.1186/s12895-018-0071-3) contains supplementary material, which is available to authorized users.

## Background

Psoriasis affects 2 to 4% of the Western population [1]. Topical calcipotriol and betamethasone dipropionate (Cal/BD) cutaneous foam is one of the safe and effective recommended first-line topical treatments of mild-to-moderate psoriasis [2, 3]. For a satisfactory treatment result, adherence to the doctor’s treatment plan prescribed for the topical treatment is considered central for the treatment result, but Storm et al. [4] reported that one out of three prescriptions were never filled at the pharmacy. According to Urquhart et al. [5], lack of efficacy from the treatment is caused by non-adherence, non-absorption or non-response. Of the three causes for lack of efficacy, the clinician may primarily influence medical adherence. This motivated our psoriasis research group to test whether one of the ubiquitous, presumably cost-effective, and easily accessible adherence-supporting technological solutions could increase adherence rates and, in addition, whether this solution reduces the severity of psoriasis and improves quality of life when tested in a high-quality and sufficiently powered randomized clinical trial (RCT).

The design of the study is based on evidence from the results of a Cochrane review [6] and a trilogy of reviews of recently-published literature by our research group [7–9], which extracted data on medical adherence rates in studies of psoriasis patients treated with topical corticosteroid compounds with an emphasis on: 1) determinants of medical non-adherence and 2) interventions that can improve rates of medical adherence.

Twelve original studies addressing adherence to topical corticosteroid treatments have been published consisting of 5 questionnaire surveys [10–14], 2 prospective studies [15, 16], 1 qualitative study [17], 1 mixed-method study [18], 1 register study [19], and 2 intervention studies [20, 21]. The observation periods varied with up to a year’s follow-up time. The rates of secondary medical non-adherence (whether prescription-only medicine picked up from the pharmacy was used) varied from 8 to 88.3% [10–12, 14–16, 18, 19, 22]. The rates of medical non-adherence were stated as follows: 1) patient-reported on eight non-validated scales [10–12, 14–16, 18, 19] and one validated scale [13], 2) objectively by a ratio of expected consumption compared with actual use (measured by the weight of the corticosteroid compound used) in two studies [15, 16], 3) objectively reported as primary non-adherence (whether written prescriptions are filled) or persistence (time from when the first prescription is filled until the end of treatment) measured via records of filled prescriptions in two studies [16, 19], and 4) number of treatment sessions measured by an electronic monitor (EM) [21]. The literature described 34 multifactorial determinants of medical non-adherence [10–12, 14, 15, 17, 18], where forgetfullness was the most frequently reported determinant [11, 15, 18]. The interventions consisted of an individualized patient-supported educational program conducted at dermatology clinics [20] and weekly patient reporting to a webpage [21]. The interventions improved medical adherence during the study periods. One of the intervention studies had a long-term follow-up period of 1 year [21]. Generally speaking, the studies were heterogeneous with a high risk of bias. The conclusions and recommendations from the literature reviews [7] and Cochrane review [6] are:

1) There is a lack of randomized, controlled clinical studies with sufficient power to test the effect of adherence-seeking interventions; 2) intervention studies can make use of technical support, e.g. apps for smartphones [23–25] or reminder messages sent to mobile telephones [26]; and 3) the formulation and exact type of the corticosteroid product should be stated.

This led us to conduct this superiority RCT, described in the following.

## Methods/design

### Study objectives

The single-blind investigator-initiated trial (Additional file 1) is an intervention study with an intention to treat (ITT) analysis that compares use of Cal/BD cutaneous foam with an EM-unit (SmarTop™ number 053776) to use of Cal/BD cutaneous foam with a patient-supporting app (MyPso SmarTop™ Version 1.0) that synchronizess via Bluetooth® to the EM unit.

### Intervention: Detailed description of the app

The EM unit and app are designed and owned by LEO® Pharma (LEO®). The app design is based on results from systematic literature reviews on adherence to topical antipsoriatic treatments [7, 8] with the goal of improving adherence rates. The app focuses on reducing forgetfullness, and at the same time incorporates functionalities that motivate (i.e. nudge) patients to use their medication [27] and thereby reduce obtrusive behaviors towards medication [28].

The patient-support app, combined with the EM unit, has three functions: 1) to provide patients with a measurement of their consumption of medicine by synchronizing to the EM (that contains a chip registering the amount of foam, day and time the patient use the foam dispenser), 2) to measure the severity of their psoriasis by having the patients state their symptoms in a diary (optional) and 3) to support patients in their treatment and refills through compulsory reminder messages that once daily pop up on the smartphone screen with a short alert sound and through use of optional educational and motivational text materials in the app.

The EM developed for the trial can only monitor the number of treatment sessions when attached to a canister with Cal/BD cutaneous foam produced and manufactured by LEO®. Since Cal/BD cutaneous foam can be expected to be more expensive than similar corticosteroid compounds, Cal/BD cutaneous foam will be supplied to all trial participants for treatment 1 hora somni (hs) pro re nata (prn) for the entire study period. The product has been approved for the Danish market since May 2016 [29].

### Recruitment

In order to ensure uniform treatment and accurate data collection, the Dermatology Department in Odense will be the only recruitment and trial site. Investigator MTS will see all participants himself. MTS will include patients referred from a planned consultation with the dermatology outpatient clinic or recruited by advertising, through local newspapers or social media platforms. If the number of participants included/year is less than 60, contact with the region’s specialists for the referral of psoriasis patients is planned.

### Null hypothesis

There is no difference in medical adherence to a topical Cal/BD product among psoriasis patients using the app (the intervention group) versus those without access to the app (the non-intervention group).

Thesis: The purpose of the trial is to test the null hypothesis.

### Endpoints and data collection

#### Primary outcome measurements

Studies should measure rates of secondary medical adherence by the following ratio: the expected quantity of topical product estimated to be used during the study period at the start of the study compared with the medicine consumed by the end of the study.

Medicinal use should be measured by an EM, a device attached to the top of the canister containing the study drug, which measures the number of treatment sessions in the treatment period [30]. In this study, we have chosen to use a study-specific EM designed by LEO®. Measuring the number of treatment sessions over the treatment period is considered superior to other methods for measuring medical adherence, such as checking the weight of the medicine used or patient-reported adherence rates.

Primary endpoint (for adherence) in week 4 is the following relationship: Number of treatment sessions with Cal/BD cutaneous foam in a 28-day treatment period/number of expected treatment sessions in a 28-day treatment period.

Data is collected from the EM on the number of treatment sessions in the treatment period. For a comparison of methods, the adherence rate reported by the participant and based on the medicine used as measured by electronic weight is also recorded in week 4.

#### Secondary outcome measurements

It should be studied whether increased consumption of prescribed medicine also results in an improvement of illness-related surrogate endpoints, which should be related to both the quality of life and severity of the disease.

For this study, the validated Lattice System Physician’s Global Assessment (LS-PGA), which combines extent, redness, scaling, and thickness of psoriasis [31–34] and the validated Dermatology Life Quality Index (DLQI), a questionnaire for how skin disease has affected the patient’s life quality in the past week up to measurement [35] have been chosen.

The secondary outcome measurements are obtained at baseline, week 4, week 8, and week 26 (see Table 1). The LS-GPA is estimated by the investigator by objective clinical investigation. The DLQI questionnaire is answered by the participants on a sheet of paper at all consultations.

**Table 1:** Enrollment, intervention, and assessment schedule

Study period							
Time points	Enrollment	Allocation	Post allocation				Close-out
	Baseline	Baseline	Baseline	Week 4	Week 8	Week 26	Week 26
Enrollment:							
Eligibility screen	X						
Informed consent	X						
Allocation		X					
Intervention arm:							
App							
Cal/BD							
Non-intervention arm:							
Cal/BD							
Assessments:							
Socio-demographics			X				
Adverse events			X	X	X	X	
Primary outcomes							
Rates of adherence:				X			
Patient report							
Weight of foam canisters							
No. treatment sessions							
Secondary outcomes							
LS-PGA			X	X	X	X	
DLQI			X	X	X	X	

Enrollment, allocation and assessments. List of abbreviations: Cal/BD: Calcipotriol/Betamethasone Dipropionate cutaneous foam; DLQI: Dermatology Quality of Life Index; BSA: Body Surface Area;LS-PGA: Lattice System Physician’s Global Assessment

#### Specification and justification of effect parameters

Electronic monitoring of secondary medical adherence is deemed to be the best quality method for measuring whether the participant has used the medicine as prescribed [8, 30]. LS-GPA is a validated outcome measurement for the estimation of the disease activity of mild-to-moderate psoriasis. DLQI is a validated outcome measurement that, over the last 15 years, has been used in the clinic and dermatological research literature.

#### Data that is considered as source data

The following source data will be recorded for the sponsor in the Case Report Form (CRF) and stored in the data program Research Electronic Data Capture (REDCap): Civil registration number, gender, age, duration of psoriasis, previous and current systemic treatments for psoriasis, local treatments for psoriasis used in the last 6 months prior to the study, socio-economic conditions (relationship status, education, job function), information as to whether the subject is pregnant or breastfeeding at the time of the study, LS-PGA and DLQI estimated in the clinic, and the test drug supplied with statement of batch numbers, the investigator’s assessment of the number of treatment sessions over 4 weeks of treatment, and the weight of the Cal/BD cutaneous foam product that is assessed as necessary for a four-week treatment period, the number of treatment days over the treatment period until the psoriasis is relieved, the number of days with psoriasis until it is relieved, the quantity of Cal/BD cutaneous foam actually used over a four-week treatment period, and the participant’s own assessment of the number of treatment sessions over a four-week treatment period.

#### Handling and storing of data

All study-relevant data will be stored in the CRF in a paper form and in REDCap. The trial master file will be stored in a printout in a binder that will be stored in a locked, fireproof cabinet at the investigator’s office.

After participants have returned the EM in week 4, it will be returned to the firm BridgeIT, which is in charge of reading the EM, and, subsequently, handed over to LEO®, which is in charge of discarding the EMs. Data from reading the EMs at BridgeIT, connected to a test-subject-unique identification number and stored in an Excel spreadsheet, will be returned by BridgeIT to the investigator. The data can in no way by BridgeIT or LEO® be linked to a civil registration number or name.

After the end of the study and after the reports have been submitted to the authorities, the sponsor will store the data at a secure, locked site for 5 years.

#### Access to source data and documents

At any time, the investigator will provide direct access to monitoring, auditing and/or inspection by the Committee on Health Research Ethics, the Danish Medicines Agency, or health authorities from other countries.

The participant’s medical chart is source data for inclusion and exclusion criteria and the progress of the treatment. The evaluation of the effect parameters (data from the EM, app, and questionnaire) will be stored in the CRF. The GCP unit will pay a monitoring visit to the trial site six times during the trial.

### Inclusion criteria

The following psoriasis patients will be recruited for this study:

- Legally competent patients of sound mind between 18 and 75 years of age
- Mild-moderate plaque and guttate type psoriasis
- The psoriasis must be visible to the investigator at the baseline visit)
- Users of smartphones (the app can be used in most types of smartphone)
- Access to a private e-mail.

During the trial, fertile women must use a reliable form of contraception, e.g. intrauterine device (IUD) or hormonal contraception (including birth-control pill, implant, transdermal contraceptive patch, vaginal ring or birth control injection), have a sterile partner, or use dual barriers during the trial period and for at least 14 days after the study ends. Prior to inclusion in the trial, evidence of a negative pregnancy test must be provided.

Breastfeeding, pregnancy, and the lack of use of reliable contraception in fertile women are exclusion criteria.

No risk for pregnant or breastfeeding women from a daily use of Cal/BD cutaneous foam at a maximal dose for 4 weeks has been reported. Nevertheless, the investigator has chosen the above-mentioned conservative method in order not to subject fertile women and any unborn fetuses to unnecessary risk.

### Exclusion criteria

The following psoriasis patients will be excluded from this study:

- Minors and patients over 75 years of age
- Legally incompetent patients or patients not of sound mind
- Patients for whom a psoriasis diagnosis cannot be objectified at the consultation
- Patients with severe psoriasis, including erythrodermic and pustular psoriasis
- Lack of possession of or ability to use a smartphone
- Breastfeeding or pregnant patients or fertile women who do not use reliable contraception
- Patients who are allergic to one of the ingredients in the Cal/BD cutaneous foam preparation.

With the installation of the app for the smartphone, the laboratory technician will ensure that: 1) the EM synchronizes to the app through Bluetooth® and 2) the app with a reminder function are set up correctly and function on the participant’s smartphone. If this is not the case, the participant will be excluded.

### Criteria for exclusion during the study

There are four criteria that must be fulfilled to be removed from the study: 1) withdrawal of consent at the last study visit in week 26, which entails that the data will not be included in the analysis from the study, 2) a serious adverse event (SAE) occurs in which a participant discontinues, 3) for fertile women, the occurrence of pregnancy, and 4) failure to keep appointments or to return the EM with legible data.

If there is a suspicion of a serious adverse event (SAE), the study’s investigator must immediately be contacted, arrange for relevant inquiries, and assess whether the test participants should cease taking the test drug.

Data that is to be collected from participants who have discontinued the study as a result of an SAE: An objective clinical evaluation of the skin will be undertaken; if hypercalcaemia or hypercalciuria has arisen, this must be confirmed by a blood test and an objective neurological investigation undertaken at the neurological department, University Hospital, Odense.

If an adrenal suppression or weakening of glycemic control of diabetes mellitus has arisen, this must be confirmed by a blood test and an objective investigation undertaken by the endocrinology department, University Hospital, Odense. If a cataract or increased intraocular pressure arises, this must be confirmed by an objective ophthalmological investigation by the ophthalmology department, University Hospital, Odense.

An attempt will be made to replace discontinued participants with new participants, so the desired total number of participants can be reached. Participants who are discontinued from the study due to the occurrence of an SAE will be followed in the relevant medical department with respect to what is estimated to be clinically relevant by the departments to which the participants are referred. Participants will be un-blinded at the time of exclusion.

### Randomization and blinding

Parallel-assigned block randomization (1:1) will be done in eight blocks stratified by age and gender.

The randomization code will be stored with the data manger and the randomization list will be made with a computer-generated sequence in the randomization tool REDCap Randomize.

While participants fill out the informed consent form, the investigator will enter participants data into the randomization tool REDCap randomize, which is set up by Odense Patient data Explorative Network (OPEN), and participants will be randomized with the help of the data program in REDCap to participate in one of the two arms of the trial.

The randomization code will be stored with the data manager, who will assist with setting up the randomization in REDCap in the OPEN system.

The trial master file (TMF) will contain information on each test participant’s EM and study drug, stating the batch numbers of the Cal/BD cutaneous foam canisters supplied.

The randomization code will be stored in the randomization tool REDCap Randomize. The trial investigator has no access to the code.

### Study drug

All study participants will be provided the study medication Cal/BD cutaneous foam (Enstilar®), registered for topical treatment of psoriasis [29]. Cal/BD cutaneous foam was chosen because the EM was design for the canister containing the Cal/BD cutaneous foam. This, in turn, could help participants focus on the testing of a new drug instead of focusing on the behavioral intervention. In the trial, both the intervention and the non-intervention groups are treated with the Cal/BD cutaneous foam, which is supplied free of charge for 28 treatment sessions (1 x daily for 4 weeks), and thereafter for 1 prn hs. Treatment will include a maximum of 15 g/day and will be supplied in an aluminum canister with an EM attached. Total consumption will be calculated at baseline: 0.5 g Cal/BD cutaneous foam per body surface area (BSA), 1× daily for 4 weeks. An agreement on the labelling of the test drug has been made with the hospital pharmacy where the study labels have been purchased and are produced in accordance with GMP (good manufacturing practice) and Annex 13 [36] in which information is provided that the trial involves a test drug. Each canister of Cal/BD cutaneous foam will be given sequentially numbered labels (starting from nos. 1, 2, 3, etc.). The study medication is listed in the TMF using this identification number.

No placebo or reference compounds will be used in the trial.

The effect on mild-to-moderate psoriasis treated daily with Cal/BD cutaneous foam for 4 weeks has been investigated in three recently published trials [37–39]. By using surrogate effect parametres for itching, life quality, and reduction in the spread of psoriasis, it was found in these studies that Cal/BD cutaneous foam was significantly more effective than treatment with either Cal/BD ointment (Daivobet®), vehicle alone, betamethasone in foam, or calcipotriol in foam. However, Cal/BD cutaneous foam was not superior to comparable products for the treatment of psoriasis of the scalp.

### Description and justification of dose level, dose regimen and frequency, and treatment period

The trial follows recommendations from the product resume for Cal/BD cutaneous foam [29]. Patients with mild-to-moderate psoriasis will be prescribed a treatment of 1 x daily for 4 weeks, thereafter according to need. Maximum consumption will be 15 g/day.

It is assessed that there are no risks connected with the use of the EM and app.

### Procedures for keeping tally of the study drug

Data entered into the TMF include a reference to the batch numbers for the medicine supplied and returned with one number for each canister of Cal/BD cutaneous foam provided, subjects identified by participant number and in the CRF, the EM-measured adherence rate (number of days with applications in the treatment period/number of days in the treatment period), and a quantity of test drug used with reference to each label number.

No placebos will be used in the study.

### Screening for side-effects

The investigator will screen for known side-effects listed in the product summary for Cal/BD cutaneous foam [29] (Additional file 2) and adhere to instructions for reporting of AEs, SAEs, serious adverse reactions (SARs), and suspected unexpected serious adverse reactions (SUSAR)s.

### Power of the study

In order for the app to increase adherence, we expect that, over the 4 weeks of the intervention period, there will be an 8% increased usage of Cal/BD cutaneous foam in the intervention group compared with the non-intervention group. Mean number of applications in the intervention group will be 90% of the recommended number of applications / 4 weeks, SD in the intervention and non-intervention groups set at 15%, power (1 – β) 80%, C.I. (two-sided) 95%, ratio sample size 1, drop-outs approx. 12.5%, total estimated test population of 134 participants.

The calculation of the required size of the test population has been assessed by the principal investigator using Stata Corp. 14.1.

### Criteria for the conclusion of the study

The study will be concluded when 120 participants have carried out the study, independent of the number of enrolled study participants with the latest inclusion of study participants to be on 28 February 2018.

### Procedures for reporting deviation from the original statistical plan

If the desired number of study participants cannot be achieved, it will be necessary to deviate from the original calculation of strength, and the level of significance (*p* <0.05) will be lowered.

### Statistical analysis

Data will be used from all the included participants who sign informed consent forms at baseline. With respect to isolated missing measurements, multiple imputations will be used. If several treatment sessions are observed on 1 day, an analysis for a maximum of 1 daily treatment session will be conducted.

When data is collected, a test of significance will be carried out using ITT analysis in regression models between means for the intervention and non-intervention groups for the outcome measures: The LS-PGA and DLQI endpoints and the difference in the overall usage of Cal/BD cutaneous foam. If individual measurements of endpoints are missing, multiple imputations will be used to compensate for the missing data.

The analyses will be conducted by a statistician at OPEN, who uses Stata Corp. 14.1 and is hired due to his experience in analyzing data from RCTs.

An interim analysis will not be conducted, since Cal/BD cutaneous foam is used in relation to recorded, approved use, and it is not assessed that there is an increased risk to the participants.

### Study plan and design

At an ordinary consultation with patients referred to the dermatology department, the investigator will screen for suitable participants according to the inclusion criteria mentioned above. Before patients are included in the study, informed consent will be obtained at the baseline visit.

### Informed consent

The investigator’s first contact with potential participants, who are legally competent and of sound mind, will take place face-to-face at an individual consultation prior to or at the baseline visit. The consultation will take place in calm surroundings at an outpatient clinic, where the investigator has no other duties at the department. Prior to presenting the information, participants will be informed of their right to make another appointment at which a companion (lay representative) is present.

The investigator is responsible for ensuring that the patient gets sufficient information about the study. This information includes oral and written information.

Oral information: this information includes the purpose, the risks and benefits of the study, possible side-effects, plan for treatment, precautions, the recording of information and duty of confidentiality, compensation and grievance options, finances, voluntariness, and informed consent and the withdrawal thereof. In addition, the patient will be informed of the possibilities for treatment if the patient does not wish to participate in the study.

Written information: Written information is provided in forms entitled “Written participant information on the study: An intervention study with the purpose of improving the use of locally-applied steroid compounds by psoriasis patients, Project ID S-20160068, Vs. 1.3” and “Rights of participants in a health-related research project,” published by the National Committee on Health Research Ethics [40].

Patients shall be made aware of their right to deliberate for 14 days and the opportunity to have any points of doubt resolved.

When the patient has decided to become a participant in the study, the patient in question must date and sign the informed consent and deliver it personally to the investigator. The informed consent form is to be dated and signed by the attesting doctor on the day the patient is informed. When the patient and investigator have signed the informed consent form, the patient must be offered a photocopy of the consent form. The original signed consent form must thereafter be stored along with the participant’s CRF.

The investigator must write in the journal that the patient has signed an informed consent and authorization form for participation in the project and the date thereof.

### Information obtained and provided throughout the study visits

At the baseline visit at the Dermatology Department in Odense, the investigator MTS will collect information from the patient journal on 1) the patient’s current and previous use of medicine, 2) length of illness, 3) socio-economic condition (marital status and educational level), 4) severity of disease, and 5) gender and age. This information alone will form the basis for a randomization control in order to ensure that the non-intervention and intervention groups are comparable.

Upon inclusion, all participants will be supplied free of charge: 1) Cal/BD cutaneous foam for 4 weeks of treatment and 2) an EM that is equipped with a canister of Cal/BD cutaneous foam from the start of the study.

No remuneration will be provided to the participants beyond the free supply of Cal/BD cutaneous foam. The Cal/BD cutaneous foam will be supplied by LEO® and delivered to the trial site.

After the consultation, participants will be shown into an adjacent room, where the laboratory assistant face-to-face will explain the treatment plan and help the participants who are randomized into the intervention group to download the app. The laboratory assistant will hand a diary for the intervention and non-intervention groups, in which the participants can write down any additional observations observed during the first 4 weeks of treatment. The participant in the intervention group will also be informed here that the app is a proposed method to help them stick to their treatment plan.

The following source data will be stored directly in the CRF:

During consultation in week 4, participants will bring the canisters containing the study drug Cal/BD cutaneous foam and the EM. The study drug will thereafter be given to the laboratory technician associated with the trial. The laboratory technician will ensure that the EM, which contains information about the number of treatment sessions in the first 4 weeks of the trial, is delivered to BridgeIT. BridgeIT will read the data and provide it to the investigator and laboratory technicians, who will transfer the data to the data storage tool REDCap (Additional file 3).

Other data from the EM on the usage pattern will be stored by the EM and the app on a data key in a locked filing cabinet with the investigator for later data processing.

LEO® will ensure that the EM will be discarded after use and will preserve no person-identifiable data from the EM.

**Fig. 1:**
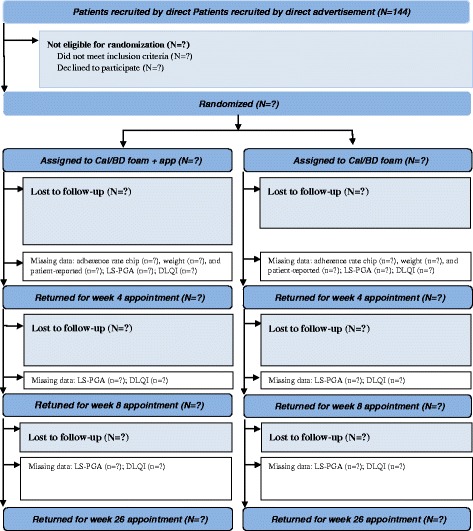
Flow of participants during the trial period

For the individual participant, the trial will last 26 weeks. All study events are listed in Table 1 and outcomes obtained at these events are listed in Fig. 1.

### Study schedule

The study included its first participant on January 9, 2017, and the last participant visit is planned for September 2017. Totally 134 participants have been included, and a total of approximately 120 participants are expected to complete the entire study period.

## Discussion

To the best of our knowledge, this is the first trial of whether an app can improve rates of adherence to a topical antipsoriatic drug. If the app improves rates of adherence and reduces the burden of psoriasis in a clinically significant way, the app could easily be implemented as a standard routine of care in the clinic.

For studies in adherence-increasing interventions, the purpose of the trial should be blinded to the participant from start of the study and during the study. Informing the participants that they are being monitored should be withheld until end of the study. This is necessary since any knowledge that the participant is being monitored may influence adherence [41]. In American studies of medical adherence, previous committees on health research ethics have approved studies in which participants were never informed that they were being monitored, and their informed consent was never given [42]; while, in another study [43], information was provided that monitoring was taking place and informed consent was given only at the final study visit. In a Danish study [16], the participatns were not informed that they were in a study that measured their medical adherence.

### Specific ethical considerations in the study

Potential beneficial effects of the use of the app: If the study can show that an app can improve adherence with respect to locally-treated psoriasis, the study will show if this is followed by a diminished extent of disease and improved quality of life.

The app developed by a commercial firm: The app developed by the pharmaceutical industry can be implemented in the clinic without cost for the public health system.

It can be seen as a disadvantage that the app was developed and is owned by the pharmaceutical industry. The public health authorities, however, will be able to draw experience from the test app and be inspired to develop similar patient-supporting apps.

Disadvantages about use of the app are that data respecting the patient’s use of the app are stored by the owner, the pharmaceutical industry. However, information about use is only connected to gender, age, and the spread of the disease without additional personally identifiable data.

It is estimated that potential advantages to the use of the app (improved medical adherence) outweigh any disadvantages about using the app.

Scientific insight achieved by the study - from an ethical perspective: Topical corticosteroids are safe and effective when they are used as prescribed. However, if the patient has a lack of efficacy from the prescribed treatment with a topical corticosteroid, it may be due to non-adherence. If the clinician is not attentive to this, the next step will be to subject the patient to treatments that have a risk of skin cancer or burns (for example, Ultaviolet B (UVB) phototherapy treatment), potentially dangerous treatments that have a risk of immunosuppression, pancytopenia and liver damage (for example, methotrexate), a high risk of extensive fetal damage, dry skin and hair loss (for example, acitretin), and financially cost-intensive treatments without knowledge of long-term side-effects (for example, biological treatments or apremilast).

## Additional files

Additional file 1: World Health Organization (WHO) Trial Registration Data Set. (PDF 384 kb)

Additional file 2: Screening and reporting of adverse events (AEs). (PDF 290 kb)

Additional file 3: Name and addresses of study site, affiliated laboratory, business partners, and public authorities providing assistance for the study. (PDF 247 kb)

## Abbreviations

AE: Adverse Event
BSA: Body Surface Area
Cal/BD: Calcipotriol and Betamethasone Dipropionate
CRF: Case Report Form
DLQI: Dermatology Quality of Life Index
EM: Electronic Monitor
EVPM: EudraVigilance Post-Authorisation Module
GMP: Good Manufacturing Practice hs Hora somni
ICH GCP: International Council for Harmonisation Good Clinical Practice
ITT: Intention To Treat
IUD: Intrauterine Device
LS-PGA: Lattice System Physician’s Global Assessment OPEN Odense Patient data Explorative Network
Prn: Pro re nata
REDCap: Research Electronic Data Capture
SAE: Serious Adverse Event
SOP: Standard Operating Procedures
SUSAR: Suspected Unexpected Serious Adverse Reaction
TMF: Trial Master File
UVB: Ultraviolet B.
