# Supplementary material for: Can an app supporting psoriasis patients improve adherence to topical treatment? A single-blind randomized controlled trial
Source: BMC Dermatol. 2018 Feb 7;18:2. doi: 10.1186/s12895-018-0071-3 (PMC5804085; doi:10.1186/s12895-018-0071-3)
Supplement: Supplementary file 1 — World Health Organization (WHO) Trial Registration Data Set. (PDF 384 kb) [file 12895_2018_71_MOESM1_ESM.pdf]

Study protocol

Can an App Supporting Psoriasis Patients Improve Adherence to Topical Treatment? A single-blind randomized controlled trial

Additional file 3: Screening and reporting of adverse events (AEs)

Local side-effects on the application site (stated from common to ordinary to very rare):

For calcipotriol

Pruritus, skin irritation, burning or stinging sensation, dry skin, erythema, rash, dermatitis, exacerbation of psoriasis, sensitivity to light, hypersensitivity reactions, angioedema, and facial edema.

For betamethasone dipropionate:

Skin atrophy, telangiectasia, striae, folliculitis, hypertrichosis, perioral dermatitis, allergic contact dermatitis, depigmentation of skin and hair, and colloid milium.

Systemic reactions – SAE:

For calcipotriol:

Hypercalcemia and hypercalciuria.

For betamethason dipropionate:

Adrenal suppression, cataracts, infections, weakening of glycemic control of diabetes mellitus, and increased intraocular pressure.

Safety evaluation

Specification and justification of safety parameters and methods and time points for measurement, recording, and analysis of safety parameters

At visits during week 4, week 8, and week 26 of the study, there will be an investigation and inquiry into side-effects. This inquiry takes its starting point in the product resume for Cal/BD foam. Any side-effects requiring treatment will be taken care of and followed-up until they are resolved and stabilized. All participants will be orally informed at baseline that, upon any suspicion of the appearance of a side-effect, they must immediately contact the investigator. Furthermore, all participants will be supplied with a business card with 24-hour direct contact information to a cell phone in the possession of the investigator, who will remain within a 2-hour distance from the University Hospital, Odense during the entire trial period.

Procedures for recording and reporting events and side-effects

The following terms for events/side-effects will be operational in the study:

Adverse Events (AE): An unwanted medical event in the participant that does not necessarily have a causal connection to the treatment with Cal/BD foam. During the study, all AEs will be observed by the investigator, who will be in charge of recording the AE in the participant's journal and CRF.

Adverse Events are classified into ordinary side-effects (cutaneous manifestations) and systemic manifestations (SAE and SUSARs (Suspected Unexpected Serious Adverse Reaction)), see listed side-effects above.

SAEs and SUSARs are systemic side-effects that include one or more of the following points: they 1) are lethal, 2) are life-threatening, 3) require hospitalization (non-elective and of at least one day's duration) or extended hospitalization, 4) result in permanent or significant invalidity, 5) result in congenital anomaly/birth defect or 6) result in other significant medical events (for example, allergic bronchospasms, cramps or affected blood tests).

All AEs that arise after the participant is included in the study will be recorded in the CRF. The following information will be recorded: 1) description of the event (toxicity and symptoms), 2) time the symptoms debuted, 3) evaluation of the degree of severity, 4) administration of treatment and solution, 5) time the condition stabilized or disappeared, and 6) relationship to the test drug.

Sponsor obligations with respect to all Serious Adverse Reactions (SARs) and SUSARs

SARs and SUSARs will be reported by the sponsor to the Committee on Health Research Ethics and the Danish Medicines Agency in accordance with applicable legislation, pursuant to which the sponsor will ensure that all lethal or life-threatening SUSARs are reported no later than 7 days after the investigator has informed the sponsor about the putative medicinal side-effect. Furthermore, the sponsor shall report no later than 8 days after the reporting of the event all relevant information about the sponsor's and the investigator's follow-up on the report to the Danish Medicines Agency.

All other SARs and SUSARS with a putative relation to the medicine shall be reported to the Danish Medicines Agency no later than 15 days after the sponsor has been made aware of them.

All reporting to the Danish Medicines Agency shall be accompanied by a statement of whether they have any consequence for the study.

Reports with respect to unexpected and putative serious side-effects (SUSARs) that are related to Cal/BD foam shall be submitted by the sponsor to the EudraVigilance Trial Module (EVCTM).

Side-effects arising in the study that are putatively related only to components other than Cal/BD foam (app and EM) and in which there is no interaction with Cal/BD foam will be reported to the EudraVigilance Post-Authorisation Module (EVPM).

Investigator's obligations with respect to SAEs and SUSARs

The investigator has the following obligations, cf. ministerial order on clinical trials: 1) the investigator shall immediately report all SAEs and SUSARs to the sponsor with respect to participants identified with a personally identifiable code number, 2) the report shall be followed up by a detailed written report to the sponsor in which the participants are once again identified with a personally identifiable code number, and 3) the

investigator shall inform the sponsor of all events and analysis results that are assessed to be critical for the safety of the participants.

All reports will be made in accordance with the rules and deadlines stated above. In the case of a death among the participants, the investigator shall submit any additional information that the sponsor requests.

The relationship of the event to the test drug is based on four degrees of causality:

1. Not related: The event is not related if exposure to the test drug has not taken place or it can be ascribed to another cause.

2. Unlikely: The event is not temporally related or it is not found likely that the event is related to the test drug.

3. Possible: the administration of the test drug and the event are reasonably temporally related and facts indicate a reasonable causality between the test drug and the event.

4. Probable: the administration of the test drug and the event are reasonably temporally related, the event can be related to the test drug more closely than it can be ascribed to other causes or the test drug is highly probably the cause.

Every conceivable medical condition that exists when a participant is screened is considered as baseline without a relation to the test drug. If the condition is exacerbated during the study, it will be reported as an AE. The highest degree of an AE will be recorded in the participant's CRF.

All medically significant AEs that are considered by the investigator or the sponsor to be related to the test drug shall be followed until they have disappeared or are assessed to be stable. Based on the investigator's clinical decision, the degree of severity of an AE may be assessed to be so pronounced that the participant must discontinue treatment. At any applicable time in the study, a participant may express a desire to discontinue treatment because of the subject's own feeling of intolerable toxicity. In both cases, the participant will be encouraged to undertake an "end-of-study assessment" (week 26) and remain under medical observation until the symptoms have ceased or the condition is stable.

Annual safety report

Once annually during the study period after the inclusion of the first participant, the investigator (the sponsor has delegated this responsibility to the investigator) will prepare a list of all serious putative side-effects that have appeared during the study period and a report on the safety of the participants. The list and report will be signed by the sponsor and sent to the Danish Medicines Agency and the Committee on Health Research Ethics. The deadline for submission of the first safety report (date of confirmation + 60 days and 1 year) will be observed.

All side-effects and events will be reported at the conclusion of the study in the final report to the Danish Medicines Agency.
