# Supplementary material for: Can an app supporting psoriasis patients improve adherence to topical treatment? A single-blind randomized controlled trial
Source: BMC Dermatol. 2018 Feb 7;18:2. doi: 10.1186/s12895-018-0071-3 (PMC5804085; doi:10.1186/s12895-018-0071-3)
Supplement: Supplementary file 2 — Screening and reporting of adverse events (AEs). (PDF 290 kb) [file 12895_2018_71_MOESM2_ESM.pdf]

Study protocol

Can an App Supporting Psoriasis Patients Improve Adherence to Topical Treatment? A single-blind randomized controlled trial

Additional file 1: World Health Organization Trial Registration Data Set.

World Health Organization Trial Registration Data Set

DATA CATEGORY	INFORMATION
Primary registry and trial identifying number	ClinicalTrials.gov, NCT 02858713
Date of registration in primary registry	August 3, 2016
Secondary identifying numbers	EudraCT number 2016-002143-42
Sources of monetary and material support	LEO® Pharma and anonymous fund
Primary sponsor	Professor Klaus Ejner Andersen, Department of Dermatology and Allergy Centre, Odense University Hospital, Denmark, e-mail: KHAndersen@health.sdu.dk
Secondary sponsor	None
Contact for public queries	Investigator, MD Mathias Tiedemann Svendsen, e-mail: mathias.tiedemann.svendsen@rsyd.dk
Contact for scientific queries	Investigator, MD Mathias Tiedemann Svendsen, e-mail: mathias.tiedemann.svendsen@rsyd.dk
Public title	Adherence in Topical Treatment of Psoriasis
Scientific title	A Designed Patient-Centered Intervention to Improve Medical Adherence in Topical Treatment of Psoriasis: A single-blind randomized controlled trial
Countries of recruitment	Denmark
Health conditions and problems studied	Medical adherence in topical treatment of psoriasis
Intervention	Intervention: Calcipotriol/betamethasone dipropionate (Cal/BD) foam + electronic monitor (EM) +application for smartphones (app)<br><br>Non-intervention: Cal/BD foam + EM
Key inclusion and exclusion criteria	Inclusion:<br><br>- Legally competent patients of sound mind between 18-75 years of age<br>- Mild-moderate plaque and guttate type psoriasis<br>- The psoriasis must be visible to the investigator at the baseline visit)<br>- Users of smartphones (the app can be used in most types of smartphone)<br>- Access to a private e-mail.

	Exclusion:<br><br>- Minors and patients over 75 years of age<br>- Legally incompetent patients or patients not of sound mind<br>- Patients for whom a psoriasis diagnosis cannot be objectified at the consultation<br>- Patients with severe psoriasis, including erythrodermic and pustular psoriasis<br>- Lack of possession of or ability to use a smartphone<br>- Breastfeeding or pregnant patients or fertile women who do not use reliable contraception<br>- Patients who are allergic to one of the ingredients in the Cal/BD foam preparation.
Study type	Interventional<br>Allocation: randomized<br>Intervention model: parallel assignment<br>Masking: Single blind (subject)<br>Primary purpose: testing an adherence improving intervention<br>Phase IV
Date of first enrolment:	January 9, 2017
Target sample size	134
Recuritment status	All participants recruited
Primary outcomes	Rates of adherence measured by:<br><br>- No treatment sessions (chip)<br>- Weight of used medication (electronic weight)<br>- Patient reported on a 4-point ordinal scale
Key secondary outcomes	DLQI: Dermatology Quality of Life Index<br>LS-PGA: Lattice System Physician's Global Assessment
