# Supplementary material for: Can an app supporting psoriasis patients improve adherence to topical treatment? A single-blind randomized controlled trial
Source: BMC Dermatol. 2018 Feb 7;18:2. doi: 10.1186/s12895-018-0071-3 (PMC5804085; doi:10.1186/s12895-018-0071-3)
Supplement: Supplementary file 3 — Name and addresses of study site, affiliated laboratory, business partners, and public authorities providing assistance for the study. (PDF 247 kb) [file 12895_2018_71_MOESM3_ESM.pdf]

Study protocol

Can an App Supporting Psoriasis Patients Improve Adherence to Topical Treatment? A single-blind randomized controlled trial

Additional file 2: General information. Name and addresses of study site, affiliated laboratory, business partners, and public authorities providing assistance for the study.

Study site

Department of Dermatology and Allergy Centre, Odense University Hospital, Kløvervænget 15, Entrance 142, DK-5000 Odense C

Pharmaceutical company that has provided a grant to pay for the study and supplied the study medication, app, and electronic monitor (EM)

LEO® Pharma, Industriparken 55, DK-2750 Ballerup

Hospital pharmacy storing and printing labels for the study medication

Hospital Pharmacy Funen, J.B. Winsløws Vej 13, Entrance 208, DK-5000 Odense C

Affiliated laboratory providing technical assistance to the study participants for the installation of the app and keeping records of electronic monitors and study medication

Dermatological Investigations Scandinavia (DIS), University of Southern Denmark, J. B. Winsløws Vej 9, DK-5000 Odense C

Company extracting data from the EM and responsible for hosting the server for the app

BridgeIT, Tobaksvejen 25, DK-2860 Søborg

Unit responsible for the randomization process and statistical analyses and providing assistance for storing data

Odense Patient data Explorative Network (OPEN), Odense University Hospital, Odense, Denmark & Department of Clinical Research, University of Southern Denmark, Odense, Denmark, J.B. Winsløws Vej 9 A, DK-5000 Odense c

Unit responsible for legally-required monitoring and audits

Good Clinical Practice (GCP) Unit, Department of Clinical Biochemistry and Pharmacology, Odense University Hospital, J. B. Winsløws Vej 19, DK-5000 Odense C
